# Supplementary material for: The evolution of negotiation strategies diversifies parental cooperation
Source: Commun Biol. 2025 Dec 4;8:1771. doi: 10.1038/s42003-025-09125-1 (PMC12706088; doi:10.1038/s42003-025-09125-1)
Supplement: Supplementary file 1 — Supplementary Information [file 42003_2025_9125_MOESM1_ESM.pdf]

Supplementary information

The evolution of negotiation strategies diversifies parental cooperation

Jia Zheng[1,2,3], Franz J. Weissing[2], Davide Baldan[4,5]

1. Ministry of Education Key Laboratory for Biodiversity Sciences and Ecological Engineering, College of Life Sciences, Beijing Normal University, China.
2. Groningen Institute for Evolutionary Life Sciences, University of Groningen, Groningen, The Netherlands
3. Institute of Ecology and Evolution, University of Bern, Bern, Switzerland.
4. Department of Biology, University of Padova, Padova, Italy
5. Department of Biology, University of Nevada, Reno, Reno, NV, U.S.A.

This Supplement consists of two parts.

Part 1: Baseline model

This part focuses on the "baseline" version of our model, where the reproductive effort of each parent is constant and not responsive to the effort of the other parent. This baseline model is a mechanistic implementation of the classical model of Houston and Davies (1985), whose fitness considerations played a central role in the evolutionary study of parental care. In **Supplementary Note 1**, we conduct a similar fitness analysis for our model and derive an analytical prediction of the evolutionarily stable parental effort. **Supplementary Figures 1 and 2** show that our simulations match the analytical predictions pretty well. Still, both the figures and the analysis reveal that the fitness considerations of Houston and Davies are less general than they appear at first sight, and they demonstrate the difficulty of capturing the evolutionary dynamics of a sex-structured population by fitness analyses.

Part 2: Robustness of the conclusions of our study

In Part 2, we conduct various sensitivity analyses of the negotiation model. **Supplementary Table 1** provides an overview of the results of these analyses. First, we check the robustness of our results regarding an increase in population size (**Supplementary Figure 3**) or changes in other model parameters (**Supplementary Figures 4 and 5**). Second, we investigate whether and to what extent the model behaviour is affected by the shape of the parental survival functions (**Supplementary Figures 6 and 7**). All additional simulations confirm our main take-home message that the evolution of parental negotiation strategies can lead to a diversity of alternative parental care patterns.

Part 1: Baseline model

Supplementary Note 1: Fitness considerations and analytical predictions

The Houston-Davies model

Our baseline model may be viewed as an individual-based implementation of the classical model of Houston and Davies (1985), a foundational contribution to parental care research. As their point of departure, Houston and Davies considered a fitness function of the form

$$W(x|y) = B(x+y) - C(x), \qquad \text{(S1)}$$

where x is the parental effort of a focal parent, y is the parental effort of its partner, $B(x+y)$ is the fitness benefit accrued via the current brood, and $C(x)$ is the fitness cost associated with the effort of the focal parent. B and C are assumed to be increasing functions, with some additional assumptions about their shape. The configuration of fitness benefits and costs is a prototype illustration of sexual conflict over parental care, as the benefits increase with the efforts of both parents, while the costs only depend on a parent's own effort. Accordingly, both parents have an (evolutionary) interest in letting the partner do most of the work. Given the fitness function (S1), Houston and Davies (and others, e.g. McNamara et al. 1999; Johnstone et al. 2014) derived conditions that must be satisfied by evolutionarily stable parental strategies and, from these, drew conclusions on the properties of these strategies.

The Houston-Davies model was a major conceptual advance, which inspired a lot of follow-up work. However, in concrete applications, determining the ingredients of the fitness function is often not straightforward. The two components are often interpreted in terms of reproductive values: $B(x+y)$ corresponds to the reproductive value of the current brood, while $C(x)$ is related to the residual reproductive value of a parent employing effort x. While there are standard methods to calculate reproductive values for several classes of models (e.g., Houston & McNamara 1999; Caswell 2001; Otto & Day 2007), these models are often "ecologically inconsistent" in that they do not include population regulation (and, hence, assume exponentially increasing or declining populations). Including population regulation in the model is important, as the mechanism of population regulation can have substantial implications for the course and outcome of evolution (e.g., Mylius & Diekmann 1995; Pen & Weissing 2000). In many examples, it may be straightforward to incorporate population regulation in the Houston-Davies model. Still, as demonstrated below, the resulting fitness function is, in these cases, often more complicated than the simple form (S1). A second complication arises because most parental care models consider the interaction between two sexes. This necessitates distinguishing between male and female reproductive values and the inclusion of the consistency requirement (the "Fisher condition") that the reproductive output of all males in the population exactly matches the reproductive output of all females (Houston & McNamara 2002; Wade & Shuster 2002; Fromhage & Jennions 2016). Although this can be done (e.g., Pen & Weissing 2002), the resulting fitness function is more complicated than equation (S1).

The take-home message is that fitness functions of the Houston-Davies type have led to considerable conceptual clarification. Still, the fitness functions of ecologically consistent two-sex models of parental care should not be expected to have the simple form (S1). As a consequence, the conclusions drawn from the Houston-Davies model cannot immediately be extrapolated to real-world scenarios.

Individual-based evolutionary models

In individual-based models, the Fisher condition is automatically satisfied. Moreover, such models must be designed to be ecologically consistent; otherwise, the modelled populations would explode. In comparison to the more standard mathematical models, individual-based evolutionary simulation models have the advantage that evolution through natural selection can be studied without specifying a fitness function (which, as in the Houston-Davies model, is often the starting point of mathematical analyses). To be sure, assumptions must be made on "fitness components" such as survival probabilities or fecundities, but these do not have to be integrated into a unified measure of "fitness".

Individual-based models have the additional advantage that the evolutionary dynamics is built into the model. To be sure, this dynamics is affected by model assumptions regarding population regulation (see above), the population size (affecting genetic drift), the mutation process (affecting diversity and the mutation-selection balance), the genetic architecture underlying the heritable strategies (affecting genetic recombination and the co-evolution of strategies), and the reproductive system (e.g., sexual versus asexual). However, these assumptions are typically more transparent than the more implicit assumptions underlying mathematical models. For example, mathematical models often assume that evolution through natural selection proceeds in the direction of the "fitness gradient", that is, in the direction of the steepest ascent of the fitness function. As shown by Long and Weissing (2023), this assumption can lead to wrong conclusions in the context of parental care evolution.

Mathematical analysis of the baseline model

Relatively simple individual-based models, like our baseline model, are, to a certain extent, amenable to mathematical analysis. As shown in Long & Weissing (2023), such analysis is useful as it sheds light on the pros and cons of the various tools used to investigate natural selection. Therefore, we here sketch the fitness analysis of our baseline model. As a first step, we have to consider population regulation. In our model, the population size is regulated because juveniles can only enter the adult population if positions become available through adult mortality. Hence, in our model, the juveniles (and not the adults) are affected by density dependence. As shown in Mylius & Diekmann (1995), this implies that "expected lifetime reproductive success" is an adequate measure of fitness.

In our model, expected lifetime reproductive success (ELRS) is given by the product of two factors: the expected number of offspring produced in a reproductive season and the expected number of reproductive seasons. As explained in the Methods section of the main text (eqn (4)), the expected number of offspring produced per reproductive season is proportional to $E_{tot}^2 \big/ (E_{tot}^2 + B)$, where $E_{tot} = x + y$ is the total effort of both parents. The expected number of

reproductive seasons of an individual employing effort x is proportional to the individual's life expectancy, which in turn is given by the inverse of the individual's per-season mortality. As in our model the probability of a parent with effort x to survive the season is $S_{par}(x) = 1 - \frac{1}{3}e^x$ (see eqn (3) in the Methods section of the main text), the life expectancy of an individual with effort x is given by: $1/(1 - S_{par}(x)) = 1/(\frac{1}{3}e^x) = 3 \cdot e^{-x}$. Taking the product of the two factors, we conclude that the ELRS of an individual employing effort x in a population where the parental partners tend to employ effort y is proportional to:

$$W(x|y) = e^{-x} \cdot \frac{(x+y)^2}{(x+y)^2 + B}. \qquad \text{(S2)}$$

While in the Houston-Davies model (S1), the reproductive costs are subtracted from the reproductive benefits, ELRS in our model corresponds to the quotient of a benefit term $(x+y)^2/((x+y)^2 + B)$ (corresponding to per-season fledgling production) and a cost term e^x (corresponding to per-season mortality). From (S2), the optimal parental effort can readily be determined. Inspection of the derivative of $W(x|y)$ with respect to x reveals that it has the same sign as the cubic

$$g(x+y) = -(x+y)^3 - B\cdot(x+y) + 2B. \qquad \text{(S3)}$$

Hence, the derivative of $W(x|y)$ with respect to x is zero if $g(x+y) = 0$, which for our default value of B ($B = 1.15$; see Methods) is solved by $x + y = 1.035$, or $x = 1.035 - y$. As the cubic g is a declining function, it must be positive to the left and negative to the right of this value of x. Accordingly, for a given value of y, the fitness function $W(x|y)$ increases for $x < 1.035 - y$ and decreases for $x > 1.035 - y$. In other words, $W(x|y)$ is maximised for $x = 1.035 - y$; this parental effort is the "best response" of an individual if its interaction partners employ effort y. From this, we can conclude that natural selection tends to achieve a total effort $x + y = 1.035$, a value that agrees reasonably well with the equilibrium total effort $S = F_m + F_f = 1.06$ we found in our simulations of the baseline model (see Fig. S1).

For parental survival functions of the form $S_{par}(x) = 1 - k\,e^x$, the evolutionarily stable parental effort is given by the solution of the equation $-(x+y)^3 - B\cdot(x+y) + 2B = 0$ and therefore only depends on the parameter B. This parameter can be interpreted in terms of offspring needs: to achieve a certain offspring survival probability, a higher total effort $E_{tot} = x + y$ is required for a larger value of B than for a lower value of B. Using the method sketched above, we also determined the evolutionarily stable parental effort for two other values of B. For $B = 0.80$, the outcome is $E_{tot} = 0.945$, and for $B = 1.50$ one obtains $E_{tot} = 1.104$. As one might expect (given the interpretation of B), the evolutionarily stable effort increases with B. Figure S2 shows that, for all three values of B considered, the simulation results agree pretty well with the analytical predictions of the equilibrium value of E_{tot}.

However, in all three cases, there is a slight but systematic discrepancy between the analytical predictions and the time average of E_{tot} in the simulations: the simulations fluctuate around somewhat larger values. This discrepancy may be explained by the fact that until now we have not considered any sex differences in the derivation of our fitness function (S2). In our model, the sexes do not differ in their life history characteristics, and the simulations in Fig. S1 reveal

that parental care is pretty egalitarian. Yet, most of the time, the difference $D = F_m - F_f$ of male and female parental efforts is not equal to zero, implying that one sex invests (slightly) less in the current brood than the other sex. This has two implications (Long et al. 2024). First, the more caring sex has a (slightly) higher mortality, reducing its life expectancy and, hence, its expected lifetime reproductive success. Second, this higher mortality results in a bias in the adult sex ratio: the less-caring sex becomes overrepresented in the population. As a consequence, not all members of the less-caring sex can find a mating partner in a given season, implying that their longer life expectancy does not result in more matings and, hence, a higher lifetime reproductive success (see Long et al. 2024 for details). All these ramifications could be incorporated into a sex-differentiated fitness function (as in Pen & Weissing 2002), but we refrain from deriving such a function in the current study.

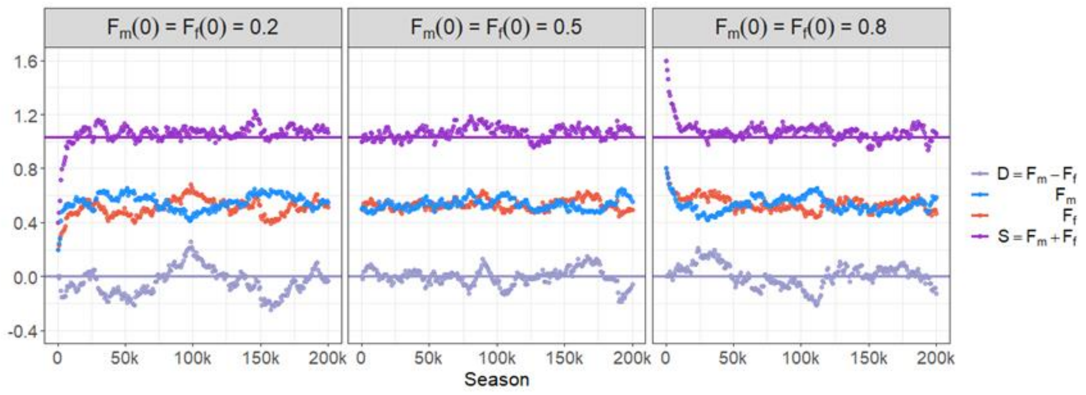

Supplementary Figure 1: Evolution of parental provisioning rates in the baseline model. In the baseline model, the parental provisioning rates (F_m and F_f for the male and female parent, respectively) do not result from negotiation but are heritable parameters that are fixed throughout an individual's lifetime (see Methods). The three panels show three representative simulations of the evolution of F_m (blue curves) and F_f (red curves) for three initial conditions of F_m and F_f. As in hundreds of other simulations, F_m and F_f rapidly converged to an equilibrium where, for our default parameters (including $B = 1.15$), both parents exhibit a provisioning rate of approximately $F_m = F_f = 0.53$ feeds per minute, resulting in a total provisioning rate of about $S = F_m + F_f = 1.06$ (purple curves). While F_m and F_f fluctuate around their equilibrium value in a complementary manner, the total provisioning rate S remains relatively constant, and it stays close to the analytically predicted value $E_{tot} = 1.035$ of the evolutionarily stable total parental effort (solid purple lines; see Supplementary Text 1 for the derivation of the predicted value). The grey curves show the difference in parental provisioning rates, $D = F_m - F_f$, which fluctuates around zero (solid grey lines). All simulations run for the baseline model resulted in egalitarian biparental care with approximately the same total provisioning level.

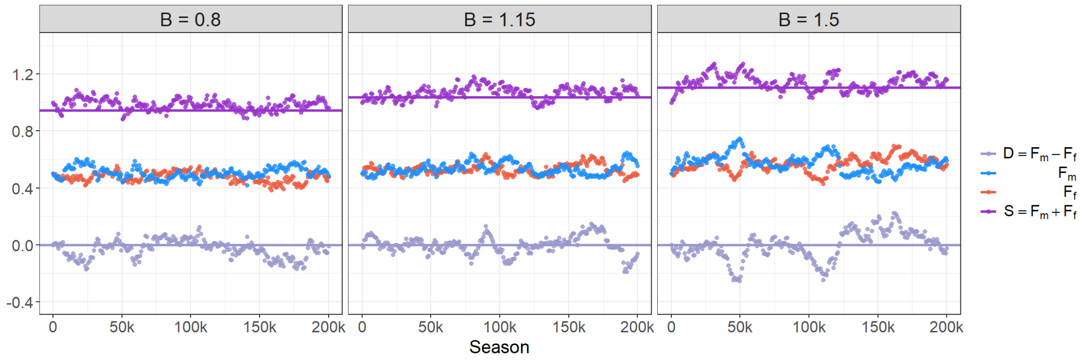

Supplementary Figure 2: Effect of model parameter *B* on evolution in the baseline model. In the baseline model, the parental provisioning rates (F_m and F_f for the male and female parent, respectively) are fixed throughout an individual's lifetime. The three panels show three representative simulations of the evolution of F_m (blue curves) and F_f (red curves) for three values of parameter *B*, which in our model fully determines the analytically predicted evolutionarily stable total provisioning rate E_{tot} of both parents (see Suppl. Text 1 for details). *B* is a parameter of the offspring survival function and can be interpreted as an indicator of the need of offspring for parental care. For the three values of *B* considered here (0.80, 1.15, and 1.50), the analytical prediction of the total parental provisioning rate is indicated by horizontal purple lines ($E_{tot}(0.80) = 0.945$, $E_{tot}(1.15) = 1.035$, and $E_{tot}(1.50) = 1.104$; see Suppl. Text 1). All simulations were initialised at $F_m(0) = F_f(0) = 0.50$. For each *B*-value, we ran 100 simulations that all converged to an equilibrium corresponding to egalitarian parental care with a total provisioning rate $S = F_m + F_f$ that is close to (but slightly larger than) the analytical prediction $E_{tot}(B)$. For the three simulations shown, the time average of the total provisioning rate *S* is $S(0.80) = 0.98$, $S(1.15) = 1.07$, and $S(1.50) = 1.13$. See Suppl. Text 1 for a potential explanation of the fact that the analytical prediction tends to be slightly off target.

Part 2: Robustness of the conclusions of our study

In Part 2, we conduct various sensitivity analyses of our model, which are described in detail and illustrated by figures below. For the reader's convenience, we begin with an overview table that summarises the main findings of the sensitivity analysis.

Supplementary Table 1. Distribution of alternative parental care patterns in eight model variants. The simulations in the main text are based on a population size of $N = 500$, and the settings of two parameters which might have a substantial effect on the evolutionary outcome: Parameter *B*, which is a measure of the offspring's need for parental care (see Suppl. Text 1), was set to $B = 1.15$, and the baseline survival of parents that do not provide care in a given season was set to $S_m(0) = S_f(0) = S_0 = \frac{2}{3}$. To check the robustness of our results, we ran numerous additional simulations for different values of either *N*, *B*, or S_0 (keeping all other parameters at the default value specified in the main text). By default, we assumed that parental survival $S(E)$ depends in a concave manner on parental effort E (given by the function $S(E) = 1 - \frac{1}{3}e^E$). We also considered the scenarios that $S(E)$ is linear or convex, and that the survival function is convex for one sex and concave for the other sex. All this resulted in eight model variants, for which the table reports the percentage of parental care patterns that evolved in 100 replicate simulations (50 simulations for the larger population size *N*).

		Percentage of simulations resulting in parental care pattern					
	Figures	egalit-arian	male-biased	female-biased	male-only	female-only	oscill-atory
Default setting (main text)	Figs 2 & 3	52%	15%	15%	6%	6%	6%
Population size 2,000 (50 simulations)	Fig. S3	70%	8%	9%	3%	4%	6%
B = 0.8 in offspring survival function	Fig. S4	21%	29%	32%	3%	4%	11%
B = 1.5 in offspring survival function	Fig. S4	52%	11%	13%	1%	2%	21%
Survival of non-caring parents *S*(0) = 0.6	Fig. S5	39%	27%	20%	3%	2%	9%
Survival of non-caring parents *S*(0) = 0.75	Fig. S5	45%	22%	19%	5%	2%	7%
Linear parental care functions	Fig. S6A	40%	24%	21%	4%	2%	9%
Convex parental care functions	Fig. S6B	3%	33%	21%	13%	15%	12%
Sex-specific shape of parental care functions	Fig. 7	0%	10%	67%	13%	0%	10%

Supplementary Note 2.1. Effect of population size *N*

By default, we focused on the population size $N = 500$ (mainly to limit the runtime of our simulations). To check whether the diversification of negotiation strategies is primarily driven by genetic drift and other forms of stochasticity in small populations, we also ran simulations for larger populations, of which we present some results for $N = 2{,}000$. Again, we identified the six alternative provisioning patterns that are shown in Fig. 3 of the main text. As illustrated in Fig. S3, the individual simulations were quite comparable with those leading to the same attractor for $N = 500$. However, as shown in Table S1, egalitarian biparental care evolved more frequently in larger than in smaller populations.

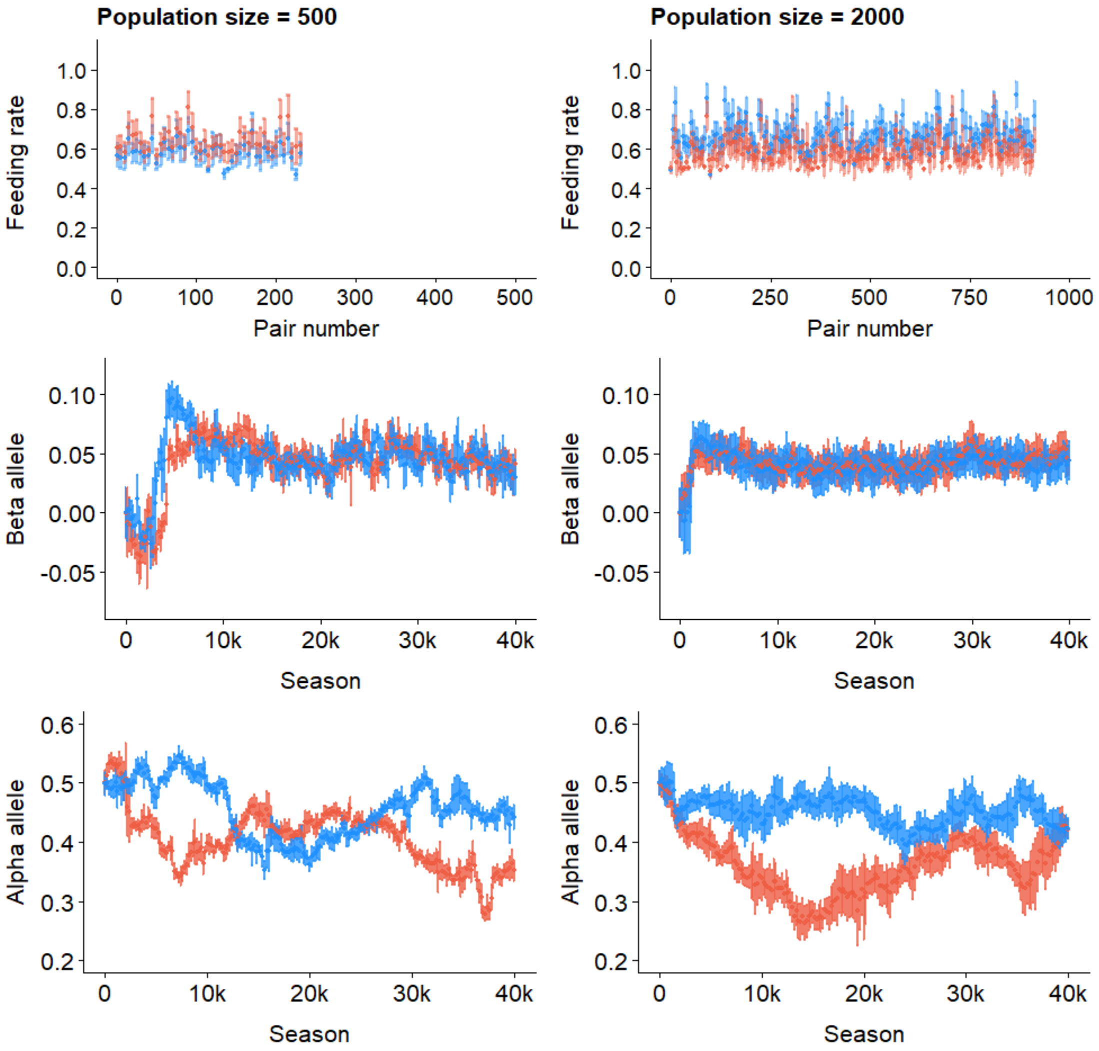

Supplementary Figure 3. Effect of population size on the evolution of parental negotiation strategies. Two representative simulations leading to egalitarian care for two population sizes: $N = 500$ (left panels) and $N = 2{,}000$ (right panels). The lower panels show the evolution of the male (blue) and female (red) β- and α-alleles (which determine the negotiation strategy) over 40k seasons. The top panels show the resulting provisioning pattern of the mated pairs in the final season (depicted as in Fig. 4 of the main text).

Supplementary Note 2.2. Effect of the offspring-need parameter *B*

In Part 1 of the Supplement, we have shown that in the baseline model (i.e., in the absence of parental negotiation), the evolutionarily stable parental provisioning rate is determined by and positively related to the offspring-need parameter *B*. In addition to our default value $B = 1.15$, we considered the values $B = 0.80$ and $B = 1.50$ (Fig. S2). Here, we did the same for the negotiation version of the model. Figure S4 (which should be compared with Fig. 2D and C of the main text) shows that the same six attractors, corresponding to six different provisioning patterns, emerge for all three *B*-values. As shown in Table S1, sex-biased provisioning evolves more often for smaller values of *B*, while egalitarian care occurs more frequently for larger *B* values.

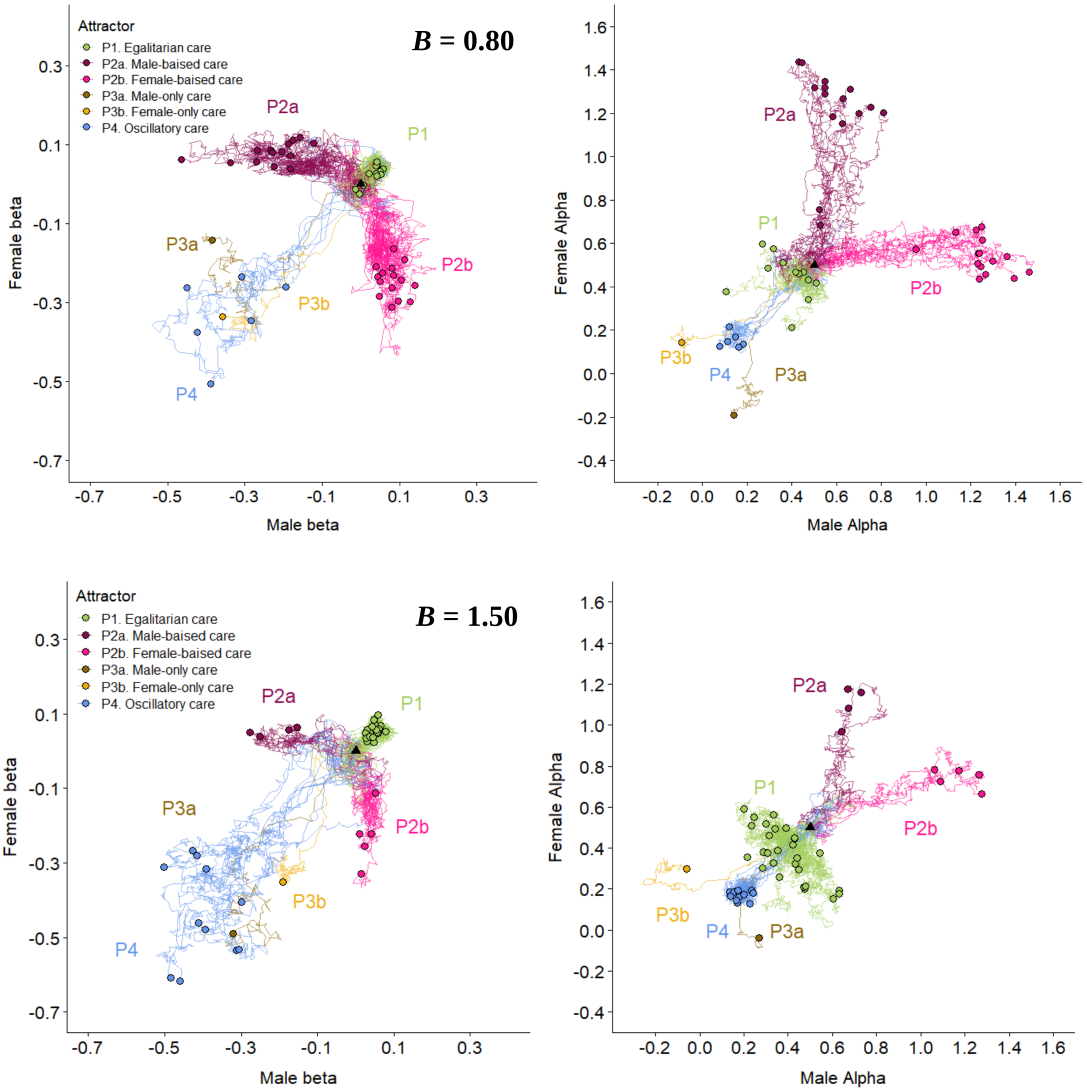

Supplementary Figure 4. Effect of parameter *B* on the evolution of parental negotiation strategies. Figure 2 in the main text shows the diversification of 100 replicate simulations for the default parameter setting, including $B = 1.15$. With the same graphical layout, we here show that the same diversification pattern is observed for two different values of *B* (top panels: $B = 0.80$; bottom panels: $B = 1.50$).

Supplementary Note 2.3. Effect of the baseline survival of parents S_0

The baseline survival of parents that do not provide care in a given season (either because they remain unmated or as a result of the negotiation process) is another important parameter of our model. In addition to the default setting $S_m(0) = S_f(0) = S_0 = \frac{2}{3}$, we here consider the two values $S_0 = 0.60$ and $S_0 = 0.75$. Figure S5 (which should be compared with Fig. 2D and C) shows that the same six attractors, corresponding to six different provisioning patterns, emerge for all three values of S_0. As shown in Table S1, egalitarian care evolved less frequently and sex-biased provisioning evolved more frequently for the lowest and the highest value of S_0, although the differences are relatively small.

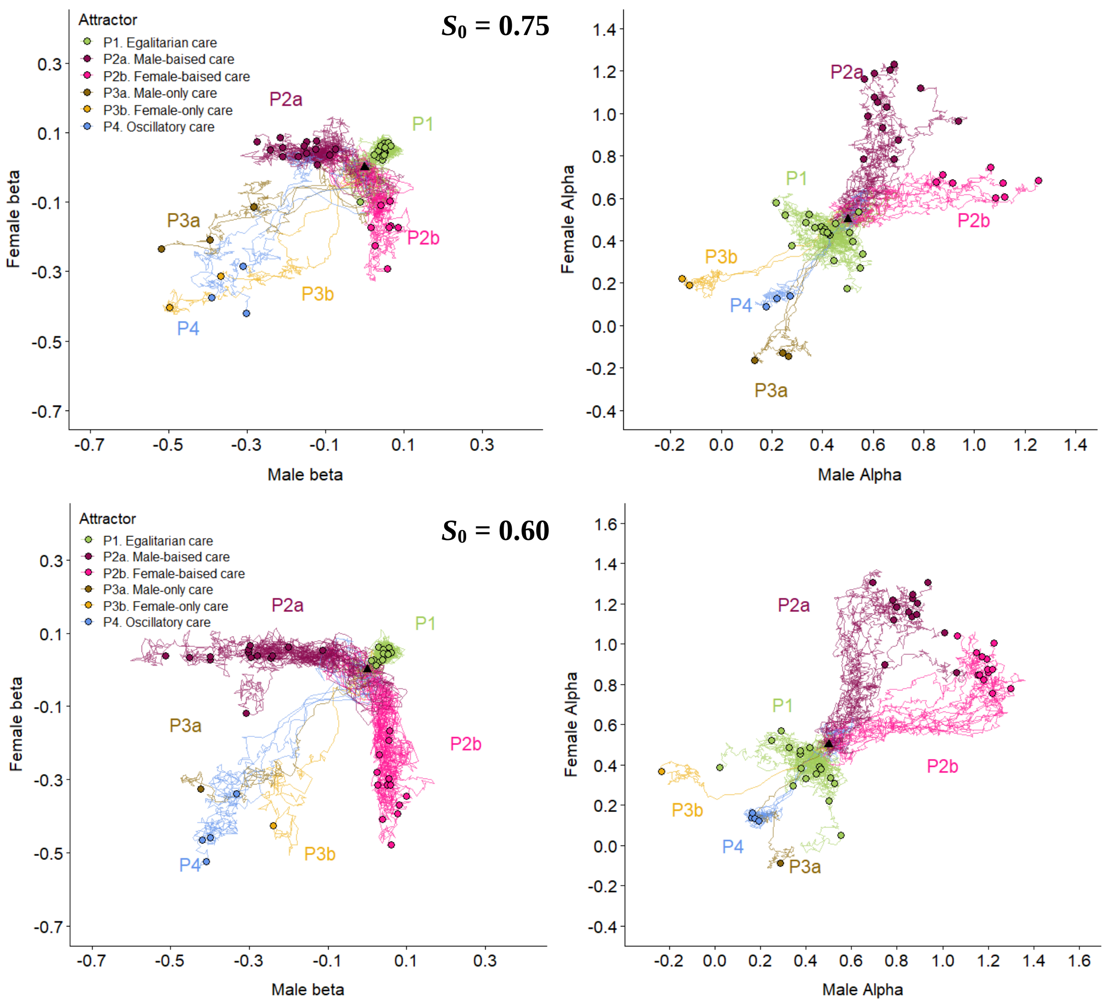

Supplementary Figure 5. Effect of parameter S_0 on the evolution of parental negotiation strategies. In the main text, we consider the parental survival functions $S(E) = 1-(1-S_0)e^E$ with baseline survival $S_0 = S(0) = \frac{2}{3}$. Figure 2 shows the diversification of 100 replicate simulations for this setting. With the same graphical layout, we show that the same diversification pattern is observed for two different values of S_0 (top panels: $S_0 = 0.75$; bottom panels: $S_0 = 0.60$).

Supplementary Note 2.4. Shape of the parental survival function

In the main text, the parental survival function was $S(E)=1-\frac{1}{3}e^{E}$ and hence a concave function of parental effort E. Here, we consider two alternative functions, which assume the same baseline survival $S_0 = S(0) = \frac{2}{3}$ and a similar survival at maximal effort $E = 1$: the linear function $S(E)=\frac{2}{3}-\frac{17}{30}\cdot E$ and the convex function $S(E)=\frac{2}{3}\cdot e^{-1.5E}$. Figure S6 shows that the same six attractors, corresponding to six different provisioning patterns, emerge for all three functions. As shown in Table S1, egalitarian care evolved most often in the case of a concave survival function and only very rarely in the case of a convex function.

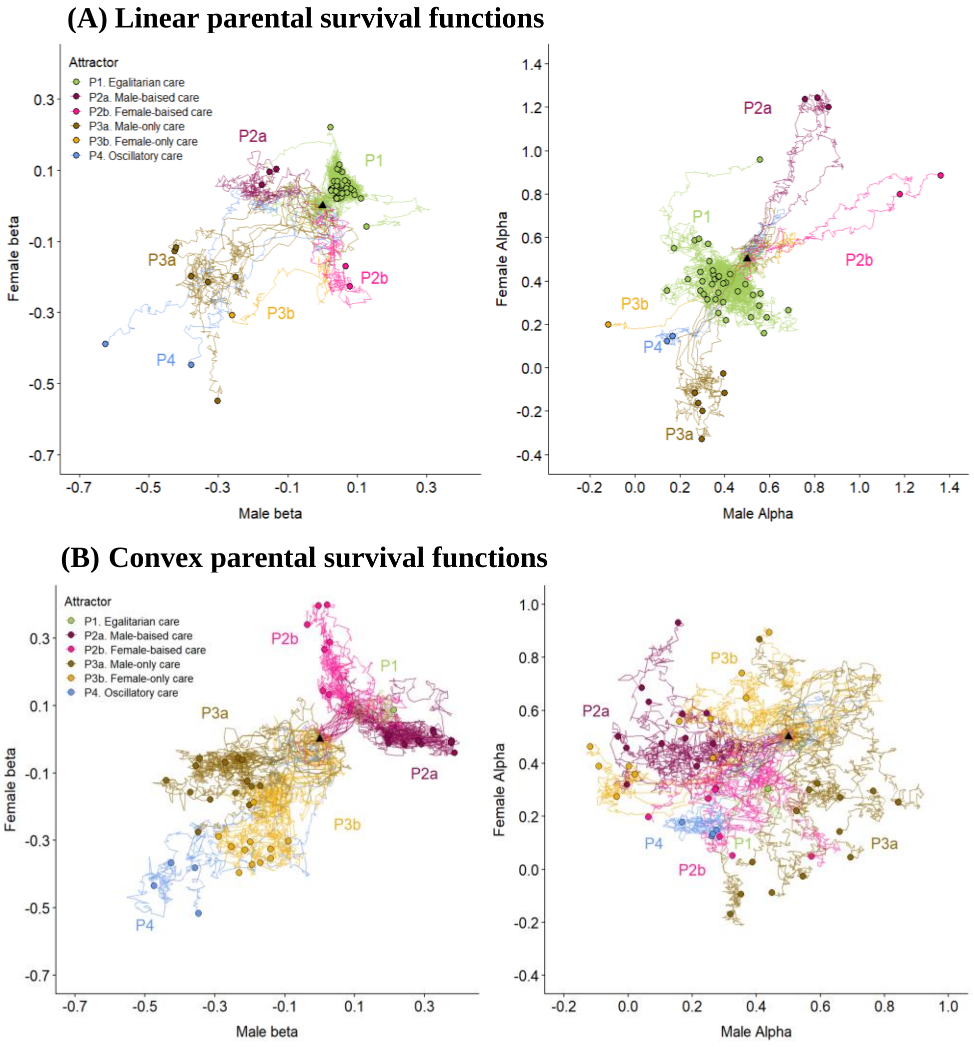

Supplementary Figure 6. Effect of survival function shape on the evolution of parental negotiation strategies. In the main text, we consider the concave parental survival functions $S(E)=1-\frac{1}{3}e^{E}$. Figure 2 shows the diversification of 100 replicate simulations for this setting. With the same graphical layout, we here demonstrate that a diversification pattern resulting in the same six attractors is also observed in the cases of linear (top panels) or convex (bottom panels) parental survival functions. However, the diversification pattern and the frequency distribution of the resulting provisioning patterns (see Table S1) differ markedly from one another.

Supplementary Note 2.5. Sex-differential parental survival function

It was one of the goals of this study to demonstrate that sex-biased parental care patterns can readily emerge from a sex-symmetric initial configuration. For this reason, we assumed that both sexes have the same parental survival functions. Figure S7 illustrates, by means of an example, how the evolutionary trajectories change if the survival function of females is concave ($S_f(E_f) = 1 - \frac{1}{3}e^{E_f}$) while the survival function of males is convex ($S_m(E_m) = \frac{2}{3} \cdot e^{-1.5E_m}$). The evolutionary outcome is still diverse, but in 100 simulations, only four alternative outcomes were observed. As shown in Table S1, two-thirds of the simulations resulted in female-biased care, while female-only care and egalitarian biparental care were not observed at all. Interestingly, 23% of the simulations resulted in male-biased or male-only care. Apparently, the difference in the shape of the survival functions impedes the evolution of egalitarian care, but it does not predestine the direction of the sex bias in care.

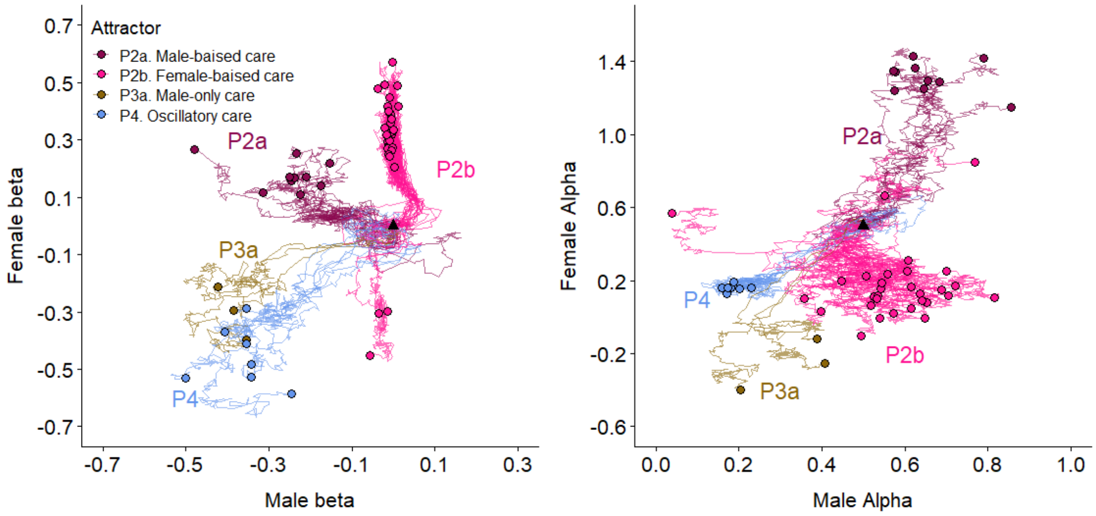

Supplementary Figure 7. Effect of a sex-differential shape of the survival function on the evolution of parental negotiation strategies. As explained in the text preceding this figure, the two panels illustrate the diversification of the parental negotiation strategies in 100 replicate simulations in a scenario where the female survival function is concave (as in the default setting of the model) while the male survival function is convex. Now, egalitarian care and female-only care are no longer observed, and the system converges to one of the four other provisioning patterns.

Supplementary references

Caswell, H. (2001) *Matrix Population Models* (2nd edition). Sinauer Associates.

Fromhage, L. & Jennions, M.D (2016) Coevolution of parental investment and sexually selected traits drives sex-role divergence. *Nature Comm.* 7, 12517.

Houston, A.I. & Davies, N.B. (1985) The evolution of cooperation and life history in the dunnock *Prunella modularis*. In: Sibly, R.M. & Smith, R.H. (eds.) *Behavioural Ecology*, pp. 471–487. Blackwell Scientific Publications.

Houston, A.I. & McNamara, J.M. (1999) *Models of Adaptive Behaviour: An Approach Based on State*. Cambridge Univ. Press.

Houston, A.I. & McNamara, J.M. (2002) A self-consistent approach to paternity and parental effort. *Philos. Trans. R. Soc. London B* 357, 351–362.

Johnstone, R.A., Manica, A., Fayet, A.L., Caswell Stoddard, M., Rodriguez-Gironés M.A. & Hinde C.A. (2014) Reciprocity and conditional cooperation between great tit parents. *Behav. Ecol.* 25: 216–222.

Long, X., Székely, T., Komdeur, J. & Weissing, F.J. (2024) A life-history perspective on the evolutionary interplay of sex ratios and parental sex roles. *American Naturalist* 205: 733457.

Long, X. & Weissing, F.J. (2023) Transient polymorphisms in parental care strategies drive divergence of sex roles. *Nature Comm.* 14, 6805.

McNamara, J. M., Gasson, C. E. & Houston, A. I. (1999) Incorporating rules for responding into evolutionary games. *Nature* 401, 368-371.

Mylius, S.D. & Diekmann, O. (1995) On evolutionarily stable life histories, optimization and the need to be specific about density dependence. *Oikos* 74, 218-224.

Otto, S.P. & Day, T. (2007) *A Biologist's Guide to Mathematical Modelling in Ecology and Evolution.* Princeton Univ. Press.

Pen, I. & Weissing, F.J. (2000) Towards a unified theory of cooperative breeding: the role of ecology and life history re-examined. *Proc. R. Soc. Lond. B* 267, 2411-2418.

Pen, I. & Weissing, F.J. (2002) Optimal sex allocation: steps towards a mechanistic theory. In: Hardy, I. (ed.): *Sex Ratios - Concepts and Research Methods*, pp. 26-45. Cambridge University Press.

Wade, M.J. & Shuster, S.M. (2002) The evolution of parental care in the context of sexual selection: a critical reassessment of parental investment theory. *Am. Nat.* 160, 285–292.
